# Secondary analysis of data on comorbidity/multimorbidity: a call for papers

**DOI:** 10.15256/joc.2015.5.57

**Published:** 2015-09-14

**Authors:** Marjan van den Akker, Jane Gunn, Stewart W. Mercer, Martin Fortin, Susan M. Smith

**Affiliations:** ^1^Department of Family Medicine, School of Public Health and Primary Care (CAPHRI), Maastricht University, Maastricht, The Netherlands; and Department of General Practice, KU Leuven, Leuven, Belgium; ^2^Primary Care Research Unit, Department of General Practice, University of Melbourne, Melbourne, Victoria, Australia; ^3^General Practice and Primary Care, Institute of Health and Wellbeing, University of Glasgow, Glasgow, UK; ^4^Department of Family Medicine and Emergency Medicine, Université de Sherbrooke, Sherbrooke, Quebec, Canada; and Centre Intégré Universitaire de santé et de services sociaux du Saguenay Lac St-Jean, Quebec, Canada; ^5^HRB Centre for Primary Care Research, Department of General Practice, RCSI Medical School, Dublin, Ireland

**Keywords:** secondary data analysis, open access, comorbidity, multimorbidity, multiple chronic conditions, chronic diseases

## Introduction

Despite the high proportion and growing number of people with comorbidity/multimorbidity, clinical trials often exclude this group, leading to a limited evidence base to guide policy and practice for these individuals [1–5]. This evidence gap can potentially be addressed by secondary analysis of studies that were not originally designed to specifically examine comorbidity/multimorbidity, but have collected information from participants on co-occurring conditions. For example, secondary data analysis from randomized controlled trials may shed light on whether there is a differential impact of interventions on people with comorbidity/multimorbidity. Furthermore, data regarding comorbidity/multimorbidity can often be obtained from registration networks or administrative data sets. These types of data sets can address a range of epidemiological research questions, such as:

- What is the prevalence and incidence of comorbidity/multimorbidity?
  - For example, the prevalence and incidence of comorbidity of cancer taken from a general practice-based registration [6]
- What are the patterns of multimorbidity?
- In the case of different data sets, cross-national comparisons of disease patterns can be performed, such as the recent analysis of primary care electronic records in Spain and the Netherlands [7]
- What is the impact of multimorbidity or specific comorbidities on patient outcomes?
  - An example includes the analysis of data from the Veterans Administration in the USA on the role of comorbidities in the prognosis of heart failure [8]. Another example is the impact of comorbid chronic conditions on the quality of diabetes care [9]
  - The Belgium BELFRAIL study, which comprised subjects aged 80 years and older, included medical histories reported by general practitioners in their database and also reported on multimorbidity as a predictor of adverse events [10]
- What is the association between multimorbidity and related phenomena?
  - For example, the Swedish study that examined the association of disability in relation to multimorbidity and disease clustering in older adults [11]
  - Another example is an Australian study examining patterns of multimorbidity in relation to functional decline [12].

Many databases include information on (chronic) diseases and are therefore available for further analysis of interesting and relevant issues related to comorbidity/multimorbidity.

## Why secondary data?

The purpose of using secondary data is to exploit the already available data and interpret them in new ways – beyond the research hypotheses for which the studies were designed. This enables researchers to explore specific questions that have not been adequately addressed and increases outputs and knowledge translation from funded research.

## Preparation and submission of secondary data analyses

The *Journal of Comorbidity* is calling for submissions of original articles based on the analysis of secondary data (i.e. existing data resources). We are interested in a broad range of research related to comorbidity/multimorbidity, and welcome secondary analyses using data from various sources, such as randomized trials and administrative, cross-sectional, and longitudinal data. Data sources may be small, such as regionally or locally based data sets, or large, nationally representative data sets.

We welcome original research of secondary analyses and invite submissions that specifically address issues relevant to comorbidity or multimorbidity. Authors should consult the journal’s website (www.jcomorbidity.com) for guidelines on preparing and submitting their paper. The journal’s usual peer review, editorial processes, and standards will be followed.
